# Adaptive Immune Dyshomeostasis as a Mediator of Vascular Cognitive Decline: Unraveling Neurovascular Crosstalk

**DOI:** 10.14336/AD.2025.0380

**Published:** 2026-06-18

**Authors:** Yating Li, Jinlin Shen, Li Zhang, Yang Luo

**Affiliations:** ^1^The First Clinical Medical School, Lanzhou University, Lanzhou, 730000 Gansu, China.; ^2^Department of Rheumatology and Immunology, The Second Hospital of Lanzhou University, Lanzhou, 730000 Gansu, China.; ^3^Department of Neurology, The First Hospital of Lanzhou University, Lanzhou, 730000 Gansu, China.; ^4^Key Laboratory of Biotherapy and Regenerative Medicine, Lanzhou 730000, Gansu, China.

**Keywords:** vascular cognitive impairment and dementia, Microglia, T cells, Th17 cells, Treg cells, B cells

## Abstract

Vascular cognitive impairment and dementia (VCID), the second most prevalent form of dementia worldwide, arises from cerebrovascular injury and is increasingly recognized as an immune-mediated neurovascular disorder. Mounting evidence implicates dysregulated immune responses—both central and peripheral—as critical drivers of VCID pathogenesis. This review highlights the pivotal roles of microglial activation, astrocytic reactivity, and infiltration of pro-inflammatory T and B cells in disrupting the neurovascular unit (NVU). These processes, mediated by cytokines such as IL-6, IL-17A, and IFN-γ, contribute to the blood-brain barrier (BBB) breakdown, white matter degeneration, and neuronal dysfunction. We further examine how systemic inflammation and comorbidities including hypertension, diabetes, and gut dysbiosis exacerbate immune-neurovascular crosstalk. In light of these insights, we discuss emerging therapeutic strategies aimed at modulating neuroimmune interactions and restoring neurovascular integrity. This integrated perspective provides a foundation for developing precise, immune-targeted interventions in the prevention and treatment of VCID.

## 1. Background

VCID encompasses a spectrum of cognitive decline and dementia, varying from mild to severe, caused by vascular brain injury and is also known as vascular dementia (VaD) [1]. In VICCCS-1 major vascular cognitive impairment category, four vascular cognitive impairment subtypes were defined: post-stroke dementia, subcortical ischemic vascular dementia, multinfarct (cortical) dementia, and mixed dementia [2]. Epidemiological research indicates that VCID ranks as the second most prevalent form of dementia following Alzheimer's disease [3]. Stroke stands out as a primary contributor to both mortality and disability across the globe and is a significant risk factor for the development of VCID [4].

In recent years, with the deepening of research, more and more evidence shows that immune system dysfunction plays a key role in the occurrence and development of VCID, and the Neurovascular Unit (NVU) is the basic structure to maintain the normal physiological function of the brain. It has become the central hub of immune-nerve-vascular interactions. The development of VCID primarily results from impaired cerebral blood flow, leading to ischemia and hypoxia, which subsequently trigger various pathological changes. These pathological insults initiate a complex immune cascade. The brain's resident immune cells, including microglia and astrocytes, become activated, while peripheral immune cells swiftly invade the affected brain area, releasing a multitude of cytokines. This cascade disrupts the body's immune functionality, leading to additional brain injury. Consequently, this results in the breakdown of BBB, demyelination of white matter, and loss of axons [3]. BBB destruction, axon loss and white matter demyelination are the core factors of VCID and may be aggravating factors. We put forward the core conceptual framework of "immune disorders-neurovascular unit injury-VCID progression", which emphasizes that the imbalance of immune homeostasis destroys the structural integrity and functional coordination of the neurovascular unit through direct or indirect effects and ultimately drives the pathological process of VCID. Clarifying the core logical relationship of "immune dysregulation, neurovascular unit injury, and VCID progress" will not only help to understand the pathological mechanism of VCID but also provide a theoretical basis for the development of innovative treatment strategies based on immune regulation.

## 2. Pathological mechanisms of VCID

The pathological process of VCID is a complex network of interwoven and synergistic effects of immune factors and various pathological mechanisms, such as Cerebral Chronic Hypoperfusion (CCH), pericyte dysfunction, BBB, and so on. Factors such as BBB destruction and diffuse white matter lesions are closely related to immune response, which jointly promote VCID progression.

CCH is recognized as one of the major pathogenic mechanisms of VCID. At the neurovascular level, a steady blood supply to the brain parenchyma is crucial for vital functions like neuronal activity, BBB maintenance, and immune cell surveillance [3]. In VCID patients, disruptions in cerebral blood flow result in various neurovascular dysfunctions, including endothelial dysfunction, glial activation, demyelination, and BBB disruption [5, 6]. Chronic reductions in cerebral blood flow within the neurovascular unit have been linked to the deterioration of white matter, the occurrence of lacunar infarctions, hemorrhaging, and brain atrophy. This includes the atrophy and loss of neurons in the hippocampus, which can ultimately lead to a decline in cognitive function [7, 8].

The neurovascular unit is a special concept that highlights the intricate relationships among different cell types in the brain. This includes mainly vascular cells such as endothelial cells, vascular smooth muscle cells, and pericytes, along with neuroglia like astrocytes, microglia, and oligodendrocytes, as well as neurons [9]. Together, these components work to uphold the integrity of BBB. which together maintain the integrity of the BBB. Additionally, the neurovascular unit plays a crucial role in regulating local blood flow in the brain, managing BBB permeability, and facilitating neuroimmune and neurovascular remodeling [10, 11]. Neurovascular unit is the core structure to maintain homeostasis in the brain, and its components are dysfunctional under the coordinated attack of immune and pathological factors. Structural and functional changes in the NVU are closely associated with altered cognitive deficits, for example, decreased density of tight junction proteins (TJP), alterations in transport proteins and cytosolic mechanisms, displacement of astrocytes, and alterations in pericyte populations can disrupt the function of the NVU, thereby exacerbating BBB disruption [12, 13].

Pericytes are multifunctional cells that encapsulate central nervous system (CNS) capillaries and help to regulate capillary diameter, vasoconstriction and diastole, capillary blood flow, and extracellular matrix protein secretion [14]. Pericytes also play an important role in immune regulation, regulating the activation of immune cells (such as T cells, macrophages and microglia) in response to proinflammatory and anti-inflammatory molecules. Pericytes also play an important role in immune regulation, regulating the activation of immune cells (such as T cells, macrophages and microglia) in response to proinflammatory and anti-inflammatory molecules [15]. It has also been shown in VCID rat models that pericellular dysfunction plays a key role in cognitive impairment [16]. CCH triggers pericyte dysfunction, and factors such as oxidative stress, inflammation, growth factor deprivation, and hypoxia can induce pericyte apoptosis, thereby impairing vascular integrity and regulation of blood flow and destroying the BBB [16].

The destruction of the BBB promotes the infiltration of peripheral immune cells, which further releases ROS and proinflammatory cytokines (such as IL-1β, IL-6, and TNF-α), thereby triggering further neuronal damage and cognitive decline [13]. After BBB is destroyed, toxic byproducts from the blood, like fibrinogen, will seep out, causing fibrinogen and fibrin to infiltrate the surrounding tissue. This process triggers the clustering and activation of macrophages and microglia, along with the recruitment and activation of chemokine T cells. Ultimately, these events contribute to axonal degeneration and a deterioration in cognitive abilities [17]. Oxidative stress arises from the overproduction of ROS, directly causing damage to neuronal function and serving as a key characteristic of VCID [18, 19].

Diffuse white matter lesion is considered to be an important pathological feature of vascular dementia. The ntegrity of white matter is crucial for facilitating smooth neuronal communication and preserving cognitive abilities [20]. When the oxygen supply of the brain is reduced, the normal function of white matter cells, especially oligodendrocytes, is subsequently destroyed, leading to demyelination, axonal degeneration and brain injury, resulting in cognitive impairment [21].

## 3. Changes in central immune cells in the VCID

The central immune landscape in VCID is characterized by complex interactions among microglia, astrocytes, and infiltrating lymphocytes, particularly CD4+ T cells. These cells, through tightly orchestrated cytokine signaling and cell-cell interactions, modulate neuro-inflammation, BBB integrity, and white matter integrity, thereby contributing to cognitive deterioration.

### 3.1 Activation and phenotypic polarization of microglia

Microglia, the primary immune cells resident in the CNS, serve as the first responders to ischemic injury. In VCID, ischemia and reperfusion trigger microglial activation, leading to profound morphological and functional changes. Resting microglia, characterized by small somata and highly branched processes, shift to an activated state marked by soma expansion and process retraction [22]. Once activated, microglia polarize into two major phenotypes: the pro-inflammatory M1 and the anti-inflammatory M2. The M1 type pumps out inflammatory agents like IL-6, IL-12, and TNF-α, which worsen neuronal damage and can push naive CD4+ T cells to transform into inflammatory Th1 and T helper 17 cells (Th17 cells) [23]. M2 microglia, by contrast, secrete IL-4, IL-10, IL-13, and TGF-β, supporting anti-inflammatory responses, tissue repair, and white matter integrity. Signals such as IL-4, IL-13, or IL-10 upregulate markers including CD206, CD163, and arginase-1, driving the M2 phenotype [24]. However, chronic inflammation promotes a shift from M2 to M1 dominance, as seen in experimental VCID models [25]. Microglia express Fc receptors (CD16, CD32) and inducible nitric oxide synthase, generating ROS and NO, which accelerate neurotoxicity [26]. Activated microglia produce MMPs that degrade the ECM and tight junction proteins, leading to BBB breakdown [27]. Perivascular microglia are recruited by endothelial chemokines and can phagocytose endothelial cells in the peri-nfarct region, exacerbating vascular rupture [28, 29]. These processes are central to VCID pathology.

**Figure 1.**
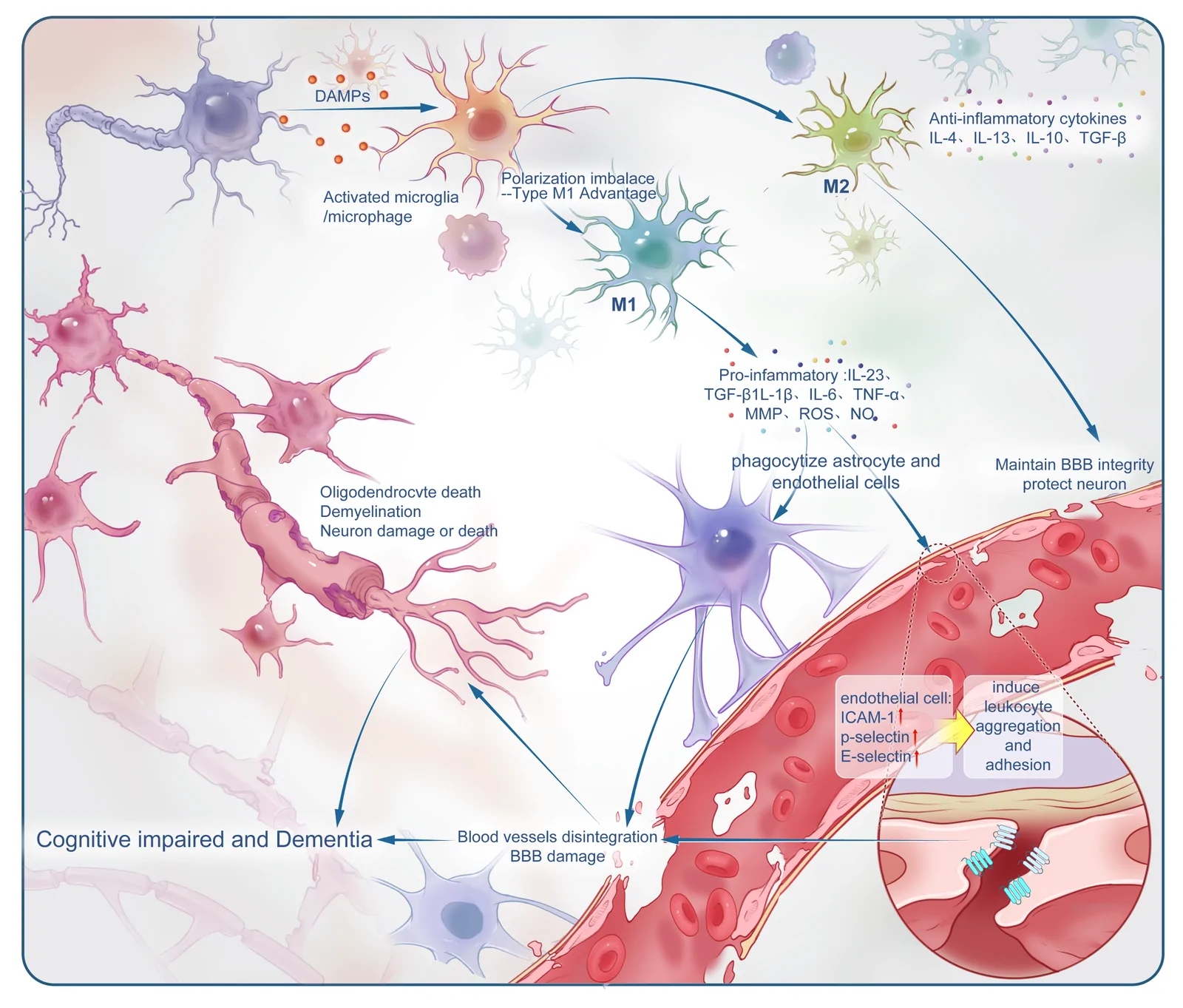
**Microglia-driven pathophysiological processes underlying VCID after brain injury**. Following brain injury, damaged neurons release DAMPs, which activate resident microglia. These microglia respond to the local microenvironment by polarizing into either a pro-inflammatory (M1) or anti-inflammatory (M2) phenotype. M2 microglia exerts anti-inflammatory and reparative effects by secreting cytokines such as IL-4, IL-10, IL-13, and TGF-β. In contrast, M1 microglia exhibits a pro-inflammatory profile, releasing cytokines including IL-1β, IL-6, TNF-α, and IL-23, as well as reactive oxygen species (ROS), nitric oxide (NO), and matrix metalloproteinases (MMPs). These mediators contribute to the disruption of the blood-brain barrier (BBB) by damaging endothelial cells and promoting the expression of adhesion molecules such as ICAM-1, P-selectin, and E-selectin on the endothelial surface. This facilitates leukocyte adhesion and transmigration, initiating a cascade of neuroinflammatory responses. Moreover, these pro-inflammatory cytokines interfere with phospholipid metabolism, leading to the production of arachidonic acid and ceramide, which further amplify inflammation and neuronal injury. M1 microglia may also phagocytose endothelial cells and astrocytes, exacerbating BBB breakdown. In addition, they can engulf myelin and axons and inhibit the maturation of oligodendrocytes, thereby impairing remyelination. These processes collectively result in white matter degeneration, oligodendrocyte death, and axonal damage, ultimately leading to neuronal loss and the clinical manifestation of cognitive impairment and dementia. This illustration spatially emphasizes these interactions—from the initial neuronal injury and DAMP release to microglial activation and polarization, endothelial damage and BBB breakdown, and finally, to white matter demyelination—all converging toward the development of vascular cognitive impairment and dementia (VCID).

Histological data demonstrate that microglial activation leads to demyelination through myelin phagocytosis and impairs oligodendrocyte maturation, especially during chronic cerebral hypoperfusion [30, 31]. Microglial depletion in VCID animal models prevents cognitive deficits, highlighting their detrimental role [32]. For instance, miR-195 downregulation in the hippocampus drives M1 polarization via the CX3CL1/CX3CR1 axis, exacerbating neurovascular damage [25]. M1 microglia also degrade astrocytic processes and axons, increase BBB permeability, and facilitate plasma protein extravasation, including fibrinogen and IgG, into the brain parenchyma [33]. CD68+ microglia-fibrinogen colocalization is correlated with dementia severity [34]. (Fig. 1)

A growing body of evidence supports the pathogenic role of microglial activation and cytokine imbalance in VCID progression. These insights provide promising therapeutic targets to modulate immune responses and preserve cognitive function in cerebrovascular disease.

### 3.2 Reactive astrocytes: key modulators of neuroinflammation and BBB integrity

Astrocytes play a crucial role in maintaining brain homeostasis by regulating ion balance, pH, neurotransmitter clearance, and the integrity of BBB [35]. In VCID, astrocytes undergo reactive transformation in response to ischemia and chronic hypoperfusion, shifting toward either a neurotoxic A1 phenotype or a neuroprotective A2 phenotype. A1 astrocytes are primarily induced by microglia-derived IL-1α, TNF-α, and complement protein C1q [36], and secrete various pro-inflammatory mediators such as IL-6, IL-17, reactive oxygen species (ROS), and glutamate. These molecules disrupt neuronal function, promote oligodendrocyte death, and increase BBB permeability [23]. In contrast, A2 astrocytes are characterized by elevated expressions of TGF-β, VEGF, and BDNF, which promote axonal regeneration and neuroprotection [35, 37]. However, in VCID, A1 astrocytes predominate, contributing to excitotoxicity, demyelination, and white matter damage, thereby accelerating cognitive decline [35]. Furthermore, reactive astrocytes influence microglial behavior through cytokine-mediated feedback loops, amplifying immune responses [38, 39].

**Figure 2.**
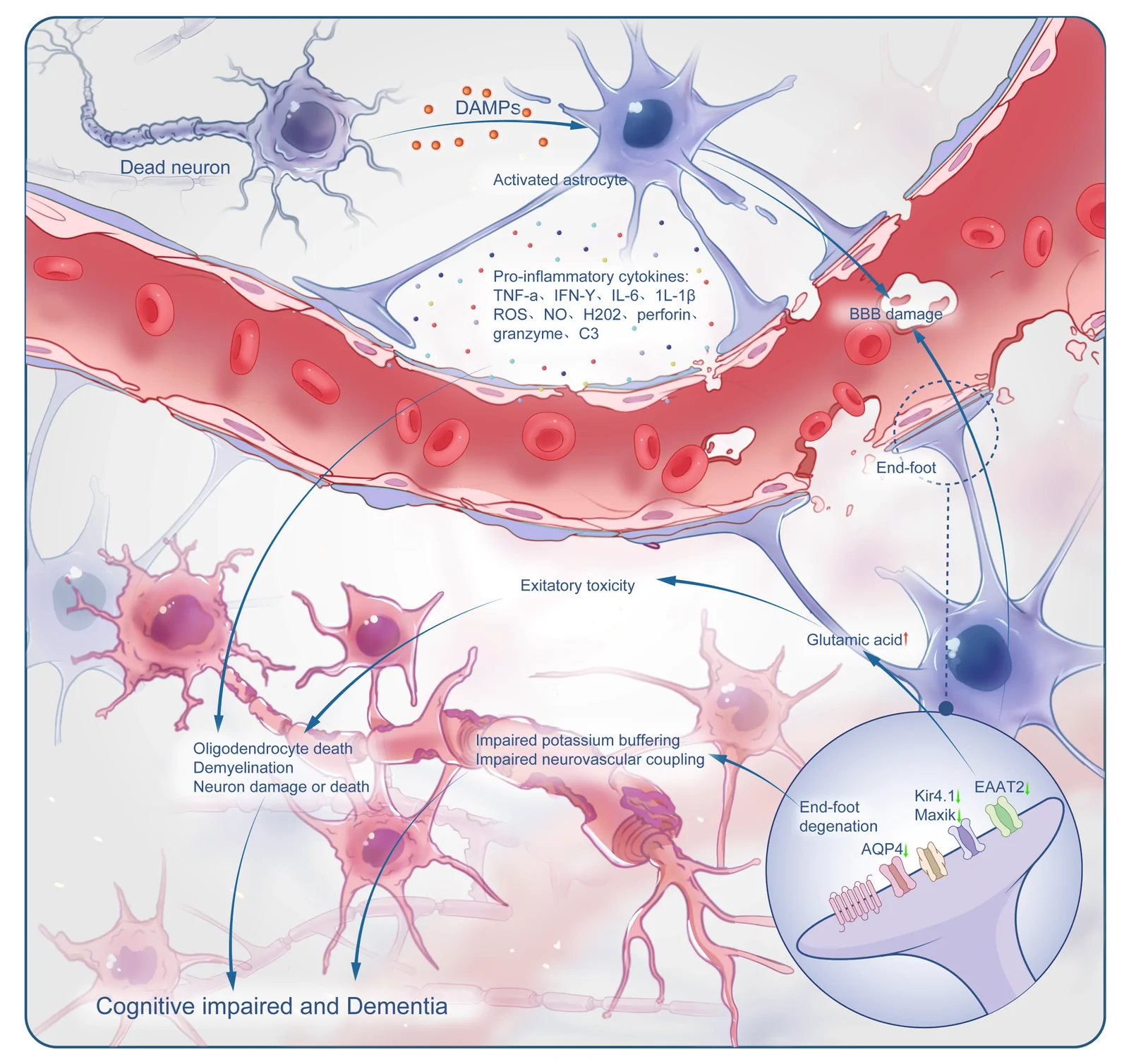
**Neuroinflammation, BBB Breakdown, and Synaptic Damage: Astrocyte Roles in VCID**. After brain injury, dying neurons release damage-associated molecular patterns DAMPs, which activate nearby astrocytes. These activated astrocytes release a variety of pro-inflammatory cytokines (TNF-α, IFN-γ, IL-6, IL-1β) and cytotoxic mediators including reactive oxygen species (ROS), nitric oxide (NO), hydrogen peroxide (H_2_O_2_), perforin, granzyme, and complement component C3. These factors compromise blood-brain barrier (BBB) integrity, leading to increased permeability and physical disruption. BBB breakdown facilitates infiltration of inflammatory molecules and cellular components, aggravating neuronal and oligodendrocyte damage, and contributing to demyelination and neurodegeneration. Astrocyte dysfunction further exacerbates pathology through degeneration of perivascular end-feet. This is characterized by the loss of anchoring protein Dp71, mislocalization of aquaporin-4 (AQP4), and reduced expression of key potassium channels (Kir4.1, MaxiK), leading to impaired potassium buffering and disrupted neurovascular coupling. The resulting elevation of extracellular potassium enhances neuronal excitability and network dysfunction. In parallel, reduced expression of excitatory amino acid transporter 2 (EAAT2) impairs glutamate clearance from the synaptic cleft, inducing excitotoxicity and synaptic injury. Together, these molecular and cellular events initiate and sustain the pathophysiological cascade of VCID, leading to cognitive decline and dementia.

Astrocytes also contribute to ionic and water homeostasis through aquaporin-4 (AQP4) and potassium channels such as Kir4.1 and MaxiK. In VCID, depolarization of AQP4, especially at perivascular endfeet, disrupts potassium regulation and impairs neurovascular coupling [40]. Matrix metalloproteinase-9 (MMP-9), released during inflammation, degrades the dystrophin-associated protein complex anchoring AQP4, further weakening BBB integrity [11]. Astrocytes regulate extracellular glutamate levels via high-affinity excitatory amino acid transporters (EAAT1 and EAAT2), thereby supporting proper neuronal excitability [41]. However, pathological remodeling of these transporters, particularly EAAT2, can lead to glutamate accumulation, oxidative stress, and cognitive dysfunction [42]. In addition, the upregulation of the Na+/H+ exchanger 1 (NHE1) in reactive astrocytes promotes the release of pro-inflammatory cytokines such as TNF-α and IL-1α, driving astrocyte proliferation and exacerbating structural damage in the hippocampus. Preclinical models of VCID demonstrate that inhibition of NHE1 or modulation of astrocytic transient receptor potential ankyrin 1 (TRPA1) signaling can alleviate cognitive deficits [38, 39]. Moreover, in a middle cerebral artery occlusion model, astrocytes were found to secrete vascular endothelial growth factor A (VEGF-A), which activates endothelial nitric oxide synthase signaling in endothelial cells, downregulates tight junction proteins such as occludin and claudin-5, and consequently increases BBB permeability, facilitating the entry of peripheral lymphocytes into the CNS [43, 44] (Fig. 2).

In summary, reactive astrocytes are central mediators in the pathogenesis of VCID. By promoting BBB disruption, sustaining neuroinflammation, and damaging white matter structure and function, they significantly contribute to cognitive impairment. Targeting astrocyte-specific signaling pathways offers a promising therapeutic strategy for preserving cognitive function in cerebrovascular disorders.

### 3.3 Cytokine Network and Signaling Integration

The progression of VCID is tightly regulated by a group of pro-and anti-inflammatory cytokines, which play a central role in neurovascular injury and repair. Among them, IL-1β, IL-6, TNF-α and TGF-β are considered to be the most critical regulators, often showing dual and even contradictory functions.

IL-1β and IL-6, typically low in healthy brains, are markedly elevated the Cerebrospinal fluid (CSF) of VCID patients [45, 46]. IL-1β, produced by microglia, astrocytes, endothelial cells, and neurons, promotes BBB leakage by downregulating ZO-1 and disrupts hippocampal synaptic plasticity [47]. In addition, IL-1β can induce BBB leakage by reducing zonula occludens-1 (ZO-1) expression in TJs [48]. Abnormal increases in IL-1β and other proinflammatory cytokines can directly lead to neuronal apoptosis, ultimately causing memory impairments in the hippocampus and disrupting synaptic plasticity, which are clinically manifested as cognitive deficits or memory disorders [48, 49]. IL-6, produced by multiple CNS cells including Th17 and γδT cells during ischemia, reduces occludin and claudin-5 expression, and skews T cell differentiation toward pro-inflammatory phenotypes [50]. IL-6 also influences the formation and equilibrium of Th17 and Regulatory T cell(Treg cells), which play a role in inflammatory disorders [51]. IL-6 has been shown to convert Treg cells into IL-17 producing cells, diminishing their inhibitory capabilities [52]. High IL-6 levels are also associated with atherosclerosis and increased infarct size [53]. Cytokines such as TNF-α, IL-1α/β, and IL-6 affect phospholipid metabolism and produce arachidonic acid-like substances, ceramides, and ROS [54]. Increased expression of these cytokines can also act on cell adhesion molecules, leading to leukocyte aggregation and adhesion, thereby inducing an inflammatory immune cascade.

TNF-α, another key cytokine, shows dual effects [55]. While it mediates inflammation and neurotoxicity, it can also regulate immune balance. Elevated peripheral TNF-α levels correlate with hippocampal atrophy and cognitive impairment. TNF-α suppresses Foxp3 expression and Treg functionality, while promoting the conversion of naive T cells into pro-inflammatory subsets [56, 57].

TGF-β is abundantly expressed by choroid plexus, astrocytes, microglia, and endothelial cells. Under physiological conditions, TGF-β regulates T cell differentiation and maintains immune tolerance by suppressing the activation and proliferation of microglia and astrocytes, thus exerting anti-inflammatory and neuroprotective effects [58, 59]. However, increasing evidence suggests that TGF-β also exhibits detrimental roles under pathological conditions. In particular, excessive or sustained TGF-β signaling promotes BBB disruption through pericyte contraction, MMP induction, and extracellular matrix remodeling [60]. Recent studies have further demonstrated that chronic overexpression of TGF-β in the aging brain or after cerebral ischemia can lead to astrocyte hypertrophy, neuronal apoptosis, and hippocampal neurodegeneration by activating Smad2/3-dependent and non-canonical signaling pathways [61, 62]. The functional outcomes of TGF-β are highly context-dependent and vary with its concentration, temporal expression, cellular origin, and the integrity of the NVU. For instance, TGF-β derived from the choroid plexus tends to preserve immune homeostasis, while astrocyte-derived TGF-β is more strongly associated with neurodegeneration in chronic VCID [63]. Thus, TGF-β represents a paradoxical mediator whose dual effects highlight the need for precise spatiotemporal modulation in potential therapeutic strategies.

VCID progression involves bidirectional regulation by cytokines IL-1β, IL-6, TNF-α, and TGF-β. IL-1β disrupts BBB integrity (via ZO-1 downregulation) and drives neuronal apoptosis; IL-6 impairs Treg function while promoting Th17 polarization, exacerbating neuroinflammation; TNF-α disrupts immune homeostasis by suppressing Foxp3 and activates NF-κB-mediated neurotoxicity; TGF-β exhibits concentration-dependent roles: protective at low doses (via microglial inhibition) but detrimental at high levels (MMP-mediated BBB disruption). These cytokines form a spatiotemporal signaling network through JAK/STAT and MAPK pathways, collectively determining the balance between neurovascular injury and repair in VCID.

## 4. Peripheral immune cell dysregulations in VCID: integrated mechanisms and clinical implications

VCID is increasingly recognized as a multifactorial condition involving not only cerebrovascular insufficiency but also chronic immune dysregulation. Accumulating evidence reveals that peripheral immune cells—including Th17, Th1, γδT, Tregs, Th2, B cells, monocytes, and NK cells—participate in a complex neurovascular immune network. Their altered activation and trafficking disrupt BBB integrity, induce neuroinflammation, and accelerate cognitive decline [64]. Cerebral hypoperfusion or ischemia upregulates hypoxia-inducible factor-1α (HIF-1α), which activates inflammatory transcription factors (e.g., NF-κB) [65], modulates immune function in myeloid-derived suppressor cells (MDSCs) and Tregs, and alters microRNA expression, promoting microglial activation and systemic immune infiltration [66, 67]. After cerebrovascular disease, T and B lymphocytes infiltrate the CNS and release a range of cytokines, such as TNF-α, IL-1β, IL-6 (proinflammatory), and IL-4, IL-10, TGF-β (anti-inflammatory), whose balance is critical in determining ischemic damage severity [68, 69].

### 4.1 Th17 and Th1 cells: drivers of neurovascular inflammation

Th17 cells play a central role in VCID progression through the release of IL-17A. Studies have shown that Th17 cells on the 3rd and 7th day after the onset of acute stroke are negatively correlated with MMSE scores at discharge [70], and the ratio of Th17 and IL-17 levels in acute stroke patients at admission is positively correlated with cognitive decline at 1 and 2 years [71]. In contrast, Th1 cells primarily release pro-inflammatory cytokines such as IL-2, IFN-γ, and TNF-α. These cytokines facilitate the conversion of microglia into M1 microglia and are key players in mediating the cellular immune response, ultimately worsening brain injury [23].

IL-17 is also released by a variety of other central and peripheral immune cells and is a proinflammatory cytokine. Microglia increased the levels of pro-inflammatory cytokines, including IL-17A, IL-23, IL-β and TNF-α, by up-regulating and activating Toll-like receptor 2 (TLR2) and sphingosine kinase 1 (Sphk1) signaling molecules at 12 hours after I/R. Astrocytes are also an important source of IL-17A release, increasing the level of IL-17A in ischemic brain tissue and cerebrospinal fluid [72]. In conclusion, the levels of central and peripheral Th17 cells and IL-17A in VCID patients are closely related to their cognitive dysfunction and are proportional to the decline of cognitive ability.

IL-17 regulates cognitive function in a variety of ways: (1) recruitment of neutrophils into the CNS; (2) damage the integrity of the BBB; (3) induction of neuronal apoptosis, hippocampal neurogenesis and neuroinflammation.

IL-17A stimulates the release of inflammatory mediators (including TNF-α, IL-6, and CXCL1) that promote neutrophil infiltration into the brain parenchyma, leading to damage in cerebrovascular disease [73]. The main effects of neutrophils are as follows: (1) they activate MMPs and hydrolyze endothelial TJs to destroy the BBB; (2) they secrete inflammatory cytokines, chemokines and adhesion molecules and induce CD8+ T cell infiltration through CXCL12; (3) they promote thrombosis. The creation of neutrophil extracellular traps can capture debris and dead cells, activating the extrinsic coagulation pathway to trigger thrombosis [74]. The expression of CXCL1, a major cytokine involved in neutrophil chemotaxis, depends on IL-17A induction [75].

During the acute stage of ischemic stroke, Th1 and Th17 cells release IFN-γ, IL-17, and IL-21, facilitating the recruitment of CD4+ T cells. It also enhances BBB permeability by disrupting TJs and reducing the levels of occludin, claudin 5, and ZO-1 [76]. IL-17A also promotes the endothelial contractile mechanism by inducing NADPH oxidase or xanthine oxidase to promote the production of ROS, leading to widening of the gap between endothelial cells, accompanied by downregulation of tightly connected molecules, thereby reducing BBB permeability [77]. In addition, through increased expression in activated microglia, IL-17 induces neuronal apoptosis, which further increases infarct size and exacerbates ischemic injury, leading to further BBB catabolism and immune cell infiltration [78].

Robust evidence indicates the involvement of TRPC6 channels in calcium homeostasis and the pivotal function of TRPC6 in neuroprotection across ischemic stroke models, both in vivo and in vitro [79]. Following a stroke, there is a swift rise in IL-17A levels, which triggers the activation of calpain to facilitate the hydrolysis of transient receptor potential cation channel 6 (TRPC6). This cascade diminishes the levels of brain-derived neurotrophic factor (BDNF) and the anti-apoptotic protein B-cell lymphoma-2 (Bcl-2), ultimately resulting in neuronal cell death [80]. Moreover, IL-17A enhances the production of proteins linked to cell death, such as caspase-3, caspase-9, and Bax, and boosts the Bax/Bcl-2 ratio post-brain trauma, effectively kickstarting the neurons' apoptosis cascade [81]. IL-17A significantly contributes to the demyelination process in the CNS [82].

In addition to mediating cognitive decline through disruption of the BBB, increasing research indicates a correlation between Th17 and IL-17A and the initiation and advancement of atherosclerosis [83], and that atherosclerosis-induced thrombosis or thromboembolism is a major pathogenetic mechanism of stroke. In addition, there is a connection between intestinal reactions and elevated peripheral IL-17 levels, particularly regarding a high-salt diet. A diet rich in salt encourages the polarization of Th17 cells by activating the p38/MAPK signaling pathway. This process results in elevated levels of IL-17 in the bloodstream, which subsequently affects brain endothelial cells, inhibiting the production of endothelial nitric oxide. The outcome is diminished cerebral blood flow and cerebrovascular issues, ultimately contributing to neuronal dysfunction and impairing cognitive abilitiesc. Furthermore, research indicates that IL-17 KO mice suffer from temporary memory lapses, while their long-term memory remains unaffected. This observation could be linked to the fact that IL-17A prompts neuroglia to secrete brain-derived neurotrophic factor (BDNF), thereby enhancing the hippocampus's neural plasticity [84]. The process by which IL-17A and Th17 cells induce cognitive impairment could also involve the suppression of hippocampal neuronal growth and neurogenesis [85].

These observations suggest that IL-17A may exhibit a dual functional profile. Under conditions of sustained inflammation or overexpression, IL-17A promotes neuroinflammation, neuronal apoptosis, and BBB disruption. However, at specific concentrations, time points, and cellular origins, IL-17A may facilitate synaptic plasticity and memory regulation by inducing neurotrophic factors such as brain-derived neurotrophic factor (BDNF) [84]. This apparent functional dichotomy may be influenced by several factors: (1) cellular source—IL-17A derived from Th17 cells versus astrocytes may act via distinct mechanisms; (2) temporal phase—acute versus chronic stages; (3) dose dependency—low levels may be modulatory while high levels are pathogenic; and (4) regional specificity—hippocampal IL-17A may enhance cognitive function, whereas cortical IL-17A may contribute to ischemic damage [84, 86].

Accordingly, targeting IL-17A as a therapeutic strategy should avoid a “one-size-fits-all” approach. Instead, intervention should be designed with spatiotemporal precision, allowing its neuroprotective roles during the acute repair phase while selectively inhibiting its pro-inflammatory effects during chronic stages. Such targeted modulation may offer critical advantages for precision immunotherapy in VCID.

IFN-γ is a cytokine that's churned out by Th1 cells, cytotoxic T cells, and NK cells. Its impact on ischemic stroke is a double-edged sword. For one, it can spark inflammation and worsen brain damage following an ischemic event. But on the flip side, it offers some level of neuroprotection and regulation, and it can influence the activation of innate nerve cells to some degree [87]. A surge in IL-6 and IFN-γ levels is linked to a more severe decline in cognitive function for VCID patients. During these abnormal states, IFN-γ is crucial for rousing microglia. It can trigger an exaggerated response from microglia, promote their growth and activation, unleash inflammatory agents and cytotoxic compounds, and ultimately result in neuronal demise [88].

### 4.2 γδT: Amplifiers of post-stroke inflammation

γδT cells are primarily localized in epithelial tissues, including the gut, where they serve as a rapid first line of defense. Although fewer in number than conventional T cells, they can rapidly secrete proinflammatory cytokines such as IL-1 and IL-17A. These cytokines recruit neutrophils and monocytes to sites of injury, contributing to the inflammatory cascade following cerebral ischemia [89]. γδ T cells also promote activation of other immune subsets including B cells, dendritic cells, αβ T cells, and NK cells, amplifying neuroinflammatory responses [90].

At homeostasis, γδ T cells are found in the meninges where they support CNS function without breaching the parenchyma [91]. However, ischemic events and gut dysbiosis can promote their activation and migration. Alterations in the intestinal microbiota following stroke shift γδ T cell polarization toward an IL-17A-producing phenotype, facilitated by inflammatory mediators such as PAMPs, IL-23, and IL-1β. These cells traverse the pia mater and infiltrate ischemic brain regions, where they aggravate injury [89, 92].

In both experimental and human studies, γδ T cells have been observed to infiltrate ischemic lesions and contribute to BBB degradation through MMP-3 and MMP-9 induction [93]. Secondaryease of IL-17A also promotes astrocyte activation and secondary cytokine production (IL-1β, IL-6), perpetuating neuro-inflammation [94]. In models of cerebral ischemia and hemorrhage, γδ T cell depletion improves motor function and reduces infarct volume and BBB damage [95, 96]. Neutralization of IL-17 significantly ameliorates cognitive and histological outcomes. Notably, γδ T cell-derived IL-17 levels exhibit a biphasic increase, peaking acutely around day 3 post-stroke and again at day 28, possibly reflecting roles in both injury and neurogenesis [97].

Taken together, γδ T cells represent a crucial link between the gut microbiome, systemic immune activation, and CNS pathology in VCID. Their dual-phase involvement suggests potential for therapeutic timing, either by suppressing early neurotoxicity or enhancing later recovery mechanisms.

### 4.3 Tregs and Th2 cells: regulators of immune homeostasis

Tregs are essential to the immune system, aiding in peripheral tolerance, modulating immune responses, and contributing significantly to immune homeostasis. Tregs exhibit a diverse range and can be classified into at least two distinct categories: natural Tregs (nTregs), which originate from the thymus, and induced Tregs (iTregs), which develop either in peripheral tissues or in vitro following stimulation with IL-2 and TGF-β [98, 99]. The combined influence of IL-2 and TGF-β is vital for the maintenance of Foxp3 expression in Tregs stimulated by TGF-β [59]. Tregs play a crucial role in modulating immune responses primarily via mechanisms reliant on immunosuppressive cytokines and antigen-presenting cells (APCs). Recent research has uncovered that iTreg subsets particularly dampen B cell activity, largely through the signaling pathways associated with TGF-β receptors [100, 101]. The naive CD4+ cells were induced to become suppressor cells by IL-2 and TGF-β, and the CD4+CD25 - cells which were subsequently cultured to become suppressor cells all expressed Foxp3 [102]. The two Treg subpopulations may have different targets or synergies in controlling unwanted immune responses [103]. In a study, iTreg cells notably suppressed TNF-α and IL-6 production from mast cells activated via a non-IgE stimulus in vitro, potentially by inhibiting NF-κB activation [104]. Recent research has shown the volatility of nTregs when exposed to specific proinflammatory cytokines, which are absent in iTregs [105].

For instance, recent reports indicate that TCR-activated nTregs can be converted into Th17 cells in vitro when IL-6 is present, which may also inhibit nTreg function [106]. TCR activation also promotes the transformation of nTregs into Th1 cells [107]. Moreover, reduced Foxp3 levels impair the regulatory role of nTregs, transforming them into Th2 cells and leading to immune disorders [108]. In the presence of proinflammatory cytokines like IL-1 and IL-6, human nTregs may be transformed into Th1 and Th17 cells [109]. In patients suffering from autoimmune maladies and other medical conditions, it's common to find elevated levels of IL-1 and IL-6. By cleverly adjusting the levels of human nTregs, we can maintain their normal phenotype and function, which in turn helps keep disease activity and progression in check [110]. Research has demonstrated, both in mice and through clinical trials, that atRA, a derivative of vitamin A, blocks the transformation of human nTreg cells into Th1 and Th17 cells. It also preserves their Foxp3 expression and suppressive abilities, whether in a test tube or within a living organism, following exposure to IL-1 and IL-6 [110, 111]. Contrary to nTregs, iTregs do not convert into Th1, Th2, Th17, or Tfh cells in inflammatory settings [112]. IL-6 and IL-1 levels are increased in patients with VCID. Therefore, whether stabilizing nTregs can alleviate the decline in cognitive function may be a new therapeutic target worth exploring. While numerous Treg subgroups have been recognized, CD4+Foxp3+ Tregs represent the most critical category, and Foxp3+ Treg cells are vital for sustaining immune balance [113]. FoxP3 is a pivotal transcription factor integral to the development and functionality of Treg cells. Prior research has shown that while a high-salt diet doesn't impact the expression of FoxP3, it actually renders the thymus-derived nTregs nonfunctional [98]. In certain autoimmune disorders, like systemic lupus erythematosus, high dietary salt intake enhances the activation and proliferation of Tfhs, B cells, plasma cells, and germinal center B cells [114].

Abnormal Treg responses within the CNS are increasingly acknowledged to trigger neuroinflammation, demyelination, and impaired function [115]. Tregs safeguard BBB integrity and thwart autoimmunity by fostering immune tolerance, suppressing other immune cell activation, or influencing brain endothelial function [116]. In stroke patients, circulating Treg levels sharply decline initially, then experience a notable and prolonged rise over the following weeks [117]. Reports indicate that VaD patients exhibit decreased peripheral Treg populations and associated atypical cytokine production [118].

In VaD model rats, we observed a considerable reduction in CD4+FoxP3+ and FoxP3+ cell populations [116] and in clinical studies, compared with healthy controls, VaD patients had reduced numbers of FoxP3+ cells [87]. The research indicates that compromised Treg activity in VCID may intensify the damage and cognitive impairments associated with ischemic stroke. Regulatory T cells, or Tregs, decrease the presence of inflammatory cytokines like TNF-α, IFN-γ, and IL-1β by increasing the production of IL-10 and TGFβ. Moreover, IL-10 and TGFβ transform CD4+ CD25- T cells into Tregs, thereby bolstering the function of these regulatory cells [102]. Following an acute ischemic stroke, the decline in CD39+ Treg cells leads to decreased levels of anti-inflammatory cytokines like TGFβ and IL-10, ultimately resulting in a diminished immune response [119]. Furthermore, Tregs regulate MMP-9 production in neutrophils via interaction with programmed death 1 (PD-1) and its ligand (PD-L1), thus improving BBB integrity [120]. Tregs have immunomodulatory effects on microglia in the ischemic brain environment [121]. The lack of Tregs spurred the shift of microglia towards the M1 phenotype. However, a surplus of Tregs encourages microglia to adopt the M2 state, thanks to the pivotal role of the IL-10/GSK3β/PTEN axis. This axis, in turn, fosters repair and the restoration of white matter function in the brain [122]. In summary, crosstalk between Tregs and M2 microglia prevents brain damage after ischemic stroke. As vascular cognitive impairment progresses, there is a notable rise in CD8+ T cells, while CD4+ Tregs experience a significant decline. This imbalance between the two cell types triggers an amplified inflammatory response, ultimately harming neurons and worsening cognitive deterioration [118]. Th17 cells and Tregs display a significant interplay, with their proliferation or function imbalances contributing to the onset of numerous infectious and immunological disorders that also correlate with VCID [123-125]. In studies involving animal models, researchers observed a reduction in inflammation by restoring the balance between Th17 and Treg cells. This process could be linked to the suppression of STAT3 and the activation of STAT5 phosphorylation, which in turn lessened cognitive impairments in rats [126]. VaD model rats exhibit dysregulated Th17/Treg homeostasis and impaired cognitive function via the modulation of Th17/Treg homeostasis [127].

**Figure 3.**
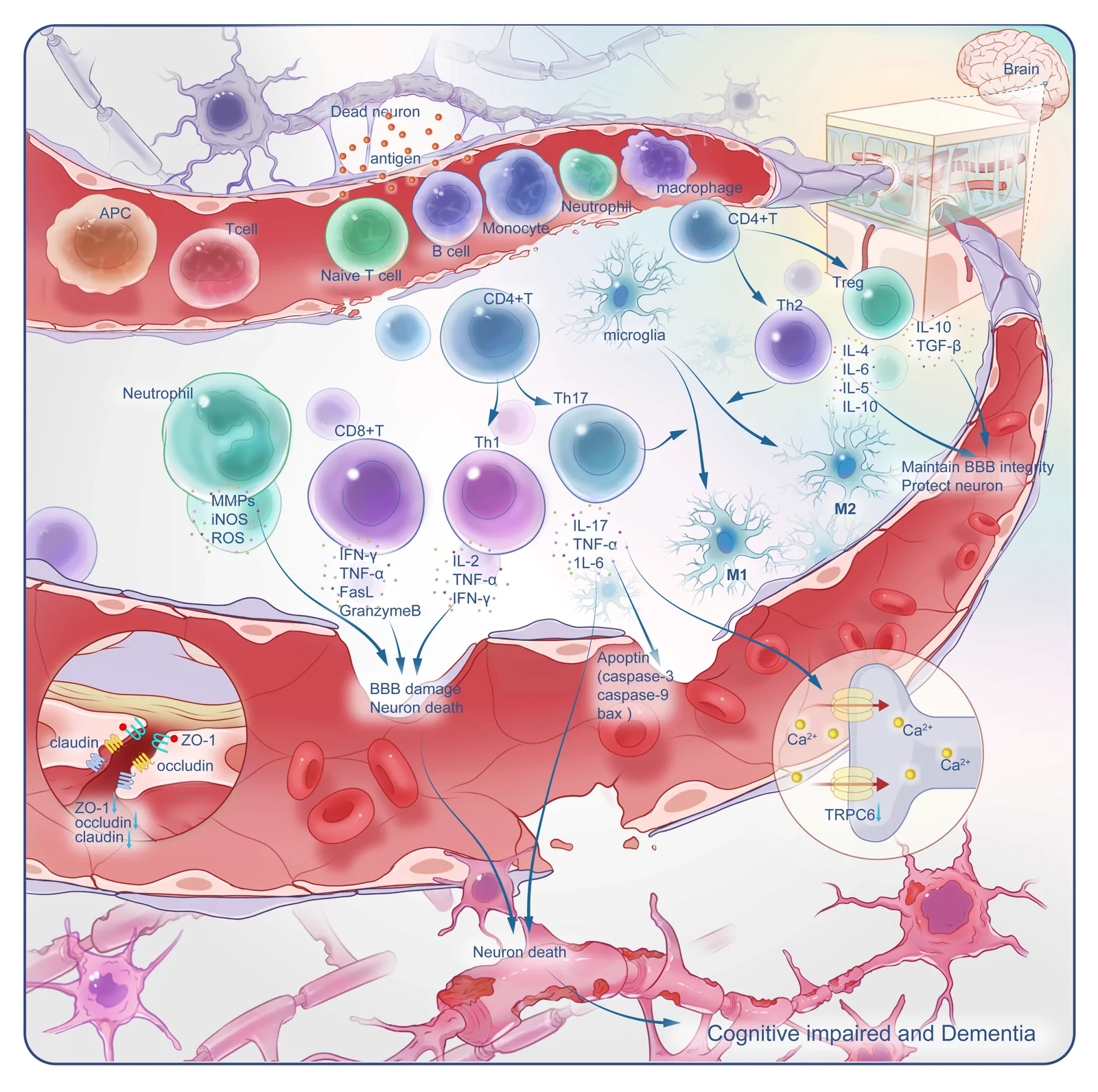
**Peripheral Immune Cell Crosstalk and Inflammatory Pathways Leading to Cognitive Decline After Brain Injury**. Following brain injury, peripheral immune cells become activated and migrate into the brain, where neutrophils produce matrix metalloproteinases (MMPs), inducible nitric oxide synthase, and reactive oxygen species (ROS), exacerbating the damage to the blood-brain barrier (BBB) and contributing to neuronal death. CD4+ T cells can differentiate into several subtypes, including Th1, Th2, Th17, and Tregs. Among these, Th1 cells release pro-inflammatory cytokines like IL-2, IL-12, TNF-α, and TFN-γ, while Th17 cells mainly produce pro-inflammatory cytokines such as IL-17, IL-6, and TNF-α. These pro-inflammatory cytokines degrade tight junctions (TJs) by reducing the expression of occludin, claudin 5, and zonula occludens-1 (ZO-1) or by directly damaging the BBB. Notably, IL-17 enhances and activates calpain to mediate the hydrolysis of transient receptor potential cation channel 6 (TRPC6), leading to decreased expression of brain-derived neurotrophic factor (BDNF) and the anti-apoptotic protein B-cell lymphoma 2 (Bcl-2), thereby resulting in neuronal death. IL-17A further promotes the expression of apoptosis-related proteins, including caspase-3, caspase-9, and Bax, initiating the neuronal apoptosis process. CD8+ cytotoxic T cells, upon antigen-dependent activation, contribute to neuronal death through cell interactions and the release of perforin and granzyme. The destruction of the BBB and neuronal apoptosis is directly or indirectly linked to vascular cognitive impairment and dementia (VCID). In contrast, Th2 and Treg cells secrete IL-4, IL-5, IL-10, and TGF-β, which exert neuroprotective effects and preserve BBB integrity. Dysregulation of the pro-inflammatory and anti-inflammatory balance of these immune cells collectively promotes the occurrence and development of cognitive dysfunction in VCID through blood-brain barrier (BBB) disruption and neuronal apoptosis pathways.

Th2 cytokines have the potential to suppress the growth of new neurons in the hippocampus by manipulating the hypothalamic-pituitary-adrenal (HPA) axis. When Th2 cells are overexpressed, they can disrupt neurogenesis and cognitive processes by boosting the levels of IL-4 in the brain and elsewhere in the body. This cytokine, IL-4, is multifaceted in its effects, influencing everything from adaptive immune responses to cognitive abilities [128]. Th2 cells produce the cytokine IL-10, a molecule that alleviates inflammation and inhibits cell apoptosis [129]. The cerebrospinal fluid IL-10 concentration in VaD patients is lower than in typical controls prior to treatment [87]. In an ischemic stroke mouse model, mice exhibiting heightened IL-10 levels displayed a larger infarct volume [130]. Clinical research indicates a correlation between diminished IL-10 levels and unfavorable stroke outcomes as well as enhanced cognitive impairments [131]. Findings indicate that the anti-inflammatory properties of IL-10 could offer a promising lead in diagnosing and treating ischemic stroke and VCID (Fig. 3).

### 4.4 B cells: mediators of autoimmunity and delayed cognitive decline.

Recent studies highlight the crucial role of B cells in post-ischemic immune responses. Although scarce in the healthy brain parenchyma, B cells infiltrate the brain rapidly after stroke, aggravating tissue damage and contributing to cognitive decline [132]. Animal models demonstrate that B cells produce IgA and IgG antibodies in the infarct area, correlating with late cognitive deficits, while B cell inhibition can mitigate these effects [133]. B cells also generate natural IgA antibodies and mount sustained responses against neuronal and myelin antigens [134, 135]. Clinically, abnormal immunoglobulin patterns and autoantibodies have been detected in the CSF and serum of stroke patients, potentially mediating neurotoxicity via Fc receptors and complement activation [136-138]. Collectively, B cell-driven autoimmunity represents a key mechanism underlying post-stroke cognitive impairment.

### 4.5 Monocytes and NK cells: peripheral effectors of neurovascular damage

Monocyte and NK cell numbers are also decreased in patients with VCID [139]. Monocyte dysfunction exacerbates neuronal loss and severe cognitive deficits in mice after cerebrovascular disease. In cerebral small vessel disease, monocytes help preserve hippocampal neurons and slow down cognitive deterioration [140]. NK and NKT cells are increased in the cerebrospinal fluid of individuals with VCID disorders [141]. NK cells are a type of innate immune cell known as cytotoxic lymphocytes. During cerebral ischemia, these cells have been noted to secrete cytokines like interferon-gamma IFN-γ, IL-10, and ROS. These substances boost the permeability of the brain's vascular endothelium, leading to the breakdown of the BBB [142].

Increased IFN-γ expression levels are accompanied by worsening cognitive deficits [118]. CD8+ T cells enhance BBB permeability and trigger neuronal apoptosis through the secretion of granzyme B, FasL, TNF-α, and IFN-γ, as well as by breaking down claudin-5 [143].

### 4.6 Translational Insights from Immune Dysregulation to Personalized Interventions in VCID

The immune dysregulation in VCID reflects a systemic imbalance between pro-inflammatory (Th17, Th1, γδT) and anti-inflammatory (Tregs, Th2) forces within the NVU, driving BBB dysfunction, immune cell infiltration, synaptic injury, and neuronal apoptosis. IL-6, IL-17A, and IFN-γ serve not only as critical pathological mediators but also as promising biomarkers for early detection, prognostic stratification, and therapeutic monitoring in VCID. For instance, elevated plasma/CSF levels of IL-17A and IL-6 at admission correlate with long-term cognitive decline after ischemic stroke [71], while increased peripheral Th17/Treg ratios or reduced FoxP3+ Treg counts predict poor outcomes [115]. Additionally, systemic inflammatory markers such as high-sensitivity C-reactive protein (hsCRP) and neutrophil-to-lymphocyte ratio (NLR) are strongly associated with VCID risk [53, 72]. Therapeutically, strategies targeting Treg stabilization (via atRA), IL-17A inhibition, or Th17/Treg axis modulation hold promise for restoring immune homeostasis [115]. Future efforts should focus on integrating longitudinal biomarker studies with immune-profiling trials to develop personalized interventions and establish a precision medicine framework for VCID management.

## 5. Relationship between the VCID and the systemic inflammatory response

Cerebrovascular diseases, such as cerebral ischemia, cause inflammation both locally and throughout the brain. When cerebrovascular diseases, dead brain cells produce damage associated molecular patterns (DAMPs), which enter the blood, DAMPs will bind to the pattern recognition receptors of peripheral immune cells, triggering systemic inflammatory response. These cells will also release cytokines when recruited into the brain, triggering neuroinflammatory cascade [144]. Neuroinflammation has the potential to disrupt neuronal function and may ultimately result in cell death, which can impair cognitive abilities. Additionally, it keeps on hammering away at the vascular system, causing more severe vascular damage and dysfunction, which can ultimately result in more profound cognitive impairments, including the development of dementia [145, 146]. Systemic inflammatory response is associated with many diseases [147] and can predict diseases, thus playing an important role in clinical diagnosis and prediction.

The lymphocyte count in the peripheral blood of patients with cognitive impairment was significantly reduced compared to healthy controls [148]. In previous reports, the number of T cells in peripheral blood and spleen of VaD model rats decreased [149], and some clinical evidence also proved that the proportion of peripheral blood T cells and CD4+ helper T cells decreased in VaD patients [139, 150]. The population of peripheral regulatory T cells (Tregs) was diminished in VaD patients, accompanied by abnormal cytokine production [118]. Cognitive impairment patients exhibited elevated neutrophil, and monocyte counts relative to lymphocytes [151]. Serum levels of highly sensitive C-reactive protein have also been shown to correlate with brain microstructure and cognitive function [152]. Research indicates a link between elevated levels of cytokines like IL-1β, IL-6, IL-8, IL-10, and IL-12 and decreased cognitive performance across multiple testing scenarios [153, 154]. Findings from cross-sectional research indicate a link between systemic inflammation, cerebrovascular disorders, white matter health, and diminished cognitive abilities [152, 155]. Research conducted by the UK Biobank developed a score for inflammatory biomarkers based on white blood cell counts and C-reactive protein levels. The findings further reinforced a significant inverse correlation between systemic inflammatory markers and cognitive performance [156]. However, contrary to this, although the inflammatory markers C-reactive protein, neutrophil/lymphocyte ratio, and white blood cell count are often elevated upon admission, they are non-specific and do not mediate the risk of cognitive impairment, whereas infection is associated with the severity of acute cognitive impairment [157]. Therefore, the connection between VCID and systemic inflammatory response needs to be further explored in order to predict VCID by systemic inflammatory response, so as to actively prevent VCID at an early stage.

## 6. Association of VCID with other diseases

### 6.1 Gut Microbiota Dysbiosis and Its Role in VCID Pathogenesis

Cerebrovascular injury can disrupt gut-brain homeostasis, leading to microbiota dysbiosis and altered microbial metabolites, such as trimethylamine N-oxide, which exacerbate cognitive decline by promoting oxidative stress, synaptic dysfunction, and inhibition of mTOR signaling [158, 159]. Elevated homocysteine, partly derived from microbial metabolism, contributes to microglial activation, BBB disruption, and periventricular white matter damage—pathologies common to VCID and other neurodegenerative diseases [160-163]. The gut microbiota shapes immune responses by regulating intestinal Tregs and Th17 cells. Tregs suppress inflammatory responses via IL-10 and TGF-β, while Th17 cells produce IL-17A and IL-22, contributing to immune-mediated CNS injury [164-166]. Restoring microbial balance through fecal transplantation or antibiotics increases Treg numbers, reduces pro-inflammatory γδT cells, and mitigates post-stroke neuroinflammation [89, 167]. Moreover, stroke-induced damage to the hypothalamic-pituitary-adrenal axis and Peyer’s patches highlights the bidirectional nature of gut-CNS immune signaling [168].

These findings underscore the gut microbiota's pivotal role as a modulator of immune-neurovascular interactions, providing a promising therapeutic target for mitigating VCID progression.

### 6.2 Hypertension-Induced Neurovascular Dysfunction in VCID

Hypertension is a major risk factor for VCID, driving white matter damage and BBB breakdown through chronic cerebrovascular remodeling [169]. Perivascular macrophages (PVMs), via Ang II type 1 receptor signaling, activate Nox2 and increase oxidative stress, leading to endothelial dysfunction and immune infiltration [170, 171]. Systemic inflammation, marked by IFN-γ and IL-1β elevation, amplifies neurovascular damage through microglial activation and synaptic loss [172, 173]. Animal models demonstrate that hypertensive injury induces white matter degeneration and cognitive deficits, while clinical studies show that even subclinical elevations in systolic blood pressure compromise white matter integrity in young individuals [174]. Although the efficacy of antihypertensive therapy on cognitive preservation remains uncertain, early blood pressure management may attenuate VCID progression [175].

Together, these data emphasize the mechanistic link between chronic vascular stress and neuroimmune activation, reinforcing hypertension as both a modifiable risk factor and a mechanistic driver of VCID.

### 6.3 Diabetes-Associated Inflammatory and Vascular Mechanisms in VCID

Type 2 diabetes mellitus (T2DM) significantly increases VCID risk, primarily through vascular amyloid deposition, lipotoxicity, and chronic low-grade inflammation [176, 177]. Hyperglycemia-induced oxidative stress disrupts the BBB, reduces cerebral perfusion, and impairs neuronal function [234]. Demyelination and neurotransmitter dysregulation in diabetic brains further exacerbate cognitive decline [177]. Diabetes intensifies post-stroke neuroinflammatory responses, with increased microglial and astrocytic activation, AQP4 polarity loss, and heightened expression of inflammatory mediators such as IL-6 and TNF-α [178-180]. TLR2-mediated innate immune activation has been linked to cognitive dysfunction and neurovascular damage in diabetic models [181]. Diabetic mice also display more severe white matter loss and neurological deficits following stroke, underscoring the importance of early cerebrovascular monitoring in diabetic patients [182].

These findings highlight diabetes not only as a risk enhancer but also as a pathological amplifier of immune-mediated neurovascular injury in VCID, underscoring the need for glycemic control in neurovascular protection strategies.

**Table 1:** Dysregulated Immune Pathways in Vascular Cognitive Impairment: Key Cellular Players, Cytokine Networks, and Therapeutic Targets.

Mechanism Category	Key Immune Cells	Major Cytokines/Signaling	Role in VCID	Evidence Sources
Central pro-inflammatory response	M1 microglia	IL-6, TNF-α, ROS, NO	Promote neuronal injury, BBB disruption, demyelination	BCCAO model, miR-195 KO
Central anti-inflammatory response	M2 microglia	IL-10, TGF-β, CD206	Tissue repair, white matter protection	Rapamycin-induced polarization
Reactive astrocytes	A1 / A2 astrocytes	IL-17, VEGF-A, MMP-9	A1: exacerbate inflammation and BBB leakage; A2: neuroprotection	MCAO model, AQP4 depolarization
Peripheral pro-inflammatory cells	Th17, Th1, γδT	IL-17A, IFN-γ, IL-21	Promote immune infiltration, neuroinflammation, neuronal death	Stroke models, gut dysbiosis
Peripheral regulatory cells	Tregs, Th2	IL-10, TGF-β, Foxp3	Suppress inflammation, maintain immune homeostasis	IL-2/atRA therapy, Foxp3 studies
B cell-mediated autoimmunity	B cells, IgG, IgA	Fc receptors, complement	Delayed neurotoxicity, autoantibody production	Rituximab model, IgG deposition
Systemic inflammatory response	Monocytes, NK cells	hsCRP, IL-6, IFN-γ	Induce BBB damage, exacerbate vascular dysfunction	CSVD models, clinical biomarkers
Comorbidity-driven pathways	Gut microbiota, hypertension, diabetes	TLRs, Nox2, HIF-1α	Amplify immune-neurovascular crosstalk	High-salt diet, diabetic models

## 7. Immune-mediated therapy in VCID

The dysregulated immune responses in VCID involve complex interactions among microglia, astrocytes, peripheral T cells, B cells, and systemic inflammation, as summarized in Table 1. Based on these mechanisms, immune-mediated therapy has emerged as a promising strategy to modulate neuroinflammation, restore neurovascular integrity, and improve cognitive function. Current research focuses on precise interventions targeting key pathways, including:

Pentoxifylline (PTX) was approved by the National Formulary of China for patients with ischemic stroke in 2010. PTX reduces the secretion of inflammatory cytokines by inhibiting the activation of microglia/macrophages in ischemic stroke models, thereby improving cognitive function [183, 184]. Clinical trials have also shown that VCID patients given PTX tend to have improved cognitive function [185], which can be used as a treatment option for VaD, but there is no international consensus. Everolimus (RAD001) can improve VaD by inhibiting mTORC1 and restoring the M1/M2 balance of microglia, which is expected to be an immunotherapy for VCID [186]. The FDA has granted Rituximab the green light as a B-cell depleting medication for conditions like rheumatoid arthritis and non-Hodgkin's lymphoma. In studies involving stroke in animals, administering Rituximab just five days post-stroke has been shown to ward off the onset of delayed cognitive decline [133]. A clinical trial revealed that natalizumab resulted in a slight improvement in cognitive function 90 days after stroke [187]. W1302 is a class 1 antivascular dementia drug developed in China and is undergoing clinical trials. Based on publicly available data, W1302 represents a novel therapeutic agent for vascular dementia. This drug primarily functions by releasing NO and inhibiting interferon-alpha (INF-α). With its unique mechanism of action, W1302 holds great promise as a potential breakthrough in the effective treatment of vascular dementia. The IL-17A/Th17 axis drives VCID inflammation, with IL-17A-targeted monoclonal antibodies like Secukinumab showing translational potential [188, 189]. Correcting the Th17/Treg imbalance using atRA or low-dose IL-2 stabilizes the Treg phenotype and reduces neuroinflammation [190]. Modulating microglial polarization with drugs such as rapamycin and PAP-1 promotes the anti-inflammatory M2 phenotype [191]. Toll-like receptors, especially TLR4, play an important role in the activation of innate immunity after stroke. ApToll, its antagonist, has entered the clinical evaluation stage and shows a good transformation prospect [192]

Future VCID treatment will evolve towards personalization and precision. Immune biomarkers such as IL-6 and IL-17A will facilitate early diagnosis and prognosis assessment. Interventions targeting the gut-brain axis, along with novel technologies including nanocarriers and Treg cell infusion, are expected to enhance therapeutic efficacy. In conclusion, immune-targeted therapy is transitioning from theoretical exploration to clinical application, opening up new avenues for precision medicine in VCID.

## 8. Discussion

VCID represents a multifactorial neurovascular disorder underpinned by chronic immune imbalance and sustained inflammation. Central immune cells—particularly microglia and astrocytes—coordinate with peripheral T cells, B cells, and cytokine networks to disrupt NVU integrity and drive cognitive decline. Th17/Treg imbalance, systemic inflammatory responses, and comorbidities such as hypertension, diabetes, and gut dysbiosis further exacerbate VCID pathology. Accumulating evidence supports the targeting of immune pathways, including IL-17A inhibition, Treg stabilization, and microglial modulation, as potential therapeutic strategies. As immunotherapy advances, precision medicine approaches integrating immune profiling and biomarker-guided interventions may offer new hope for VCID prevention and treatment. Future research should prioritize longitudinal studies and clinical trials to validate these immune-based strategies and elucidate their translational relevance.
